# Day and Night GSH and MDA Levels in Healthy Adults and Effects of Different Doses of Melatonin on These Parameters

**DOI:** 10.1155/2011/404591

**Published:** 2011-04-28

**Authors:** Shilpa Chakravarty, Syed Ibrahim Rizvi

**Affiliations:** Department of Biochemistry, University of Allahabad, Allahabad 211002, India

## Abstract

The pineal secretory product melatonin (chemically, N-acetyl-5-methoxytryptamine) acts as an effective antioxidant and free-radical scavenger and plays an important role in several physiological functions such as sleep induction, immunomodulation, cardiovascular protection, thermoregulation, neuroprotection, tumor-suppression and oncostasis. Membrane lipid-peroxidation in terms of malondialdehyde (MDA) and intracellular glutathione (GSH) is considered to be a reliable marker of oxidative stress. The present work was undertaken to study the modulating effect of melatonin on MDA and GSH in human erythrocytes during day and night. Our observation shows the modulation of these two biomarkers by melatonin, and this may have important therapeutic implications. *In vitro* dose-dependent effect of melatonin also showed variation during day and night. We explain our observations on the basis of melatonin's antioxidative function and its effect on the fluidity of plasma membrane of red blood cells. Rhythmic modulation of MDA and GSH contents emphasized the role of melatonin as an antioxidant and its function against oxidative stress.

## 1. Introduction

Oxidative stress, or the imbalance between oxidant production and antioxidant levels, appears to be favour of the former, consequently resulting in acceleration of neurodegeneration, cognitive impairment, immunosuppression, and ageing [1]. Melatonin (N-acetyl-5-methoxytryptamine) has been known for a long time as the major hormone produced by the pineal gland, but later, it has emerged as a compound that can also be synthesized in other organs and tissues and serves as an autacoid factor. Pineal melatonin is involved in many physiological functions, among them sleep promotion, circadian regulation, immunomodulation, neuroprotection, and tumour suppression. This pineal indoleamine exhibits characteristic diurnal rhythm of synthesis and secretion, which attains its peak at night followed by a gradual decrease during the daytime. In addition, pharmacological doses of melatonin effectively reduce oxidative stress through a number of mechanisms [2]. 

Melatonin scavenges hydrochlorous acid, detoxifies highly toxic hydroxyl and peroxyl radicals *in vitro* and scavenges peroxynitrite. It has also been reported to increase the synthesis of glutathione and of several antioxidant enzymes [3]. Upon metabolism, melatonin converts to a number of antioxidant compounds such as, N^1^-acetyl-N^2^-formyl-5-methoxy-kynuramine and N^1^-acetyl-5-methoxy-kynuramine [4]. Therefore, melatonin is considered to be a broad-spectrum antioxidant. It was found to be more powerful than glutathione and mannitol in neutralizing free radicals and can protect cell membranes from oxidative damage more effectively than vitamin E [1, 5].

 The present study was undertaken to understand the modulation of intracellular reduced glutathione (GSH) and malondialdehyde (MDA) by melatonin in human red blood cells according to the oscillatory circadian changes in levels of this hormone. We have also studied the dose-dependent effect of melatonin on GSH and MDA in erythrocytes obtained from blood at two different times, subjected to oxidative stress by incubating with tert-butyl hydroperoxide (t-BHP) [6]. We used t-BHP as pro-oxidant, because it does not undergo degradation by the cytosolic catalase [7]. Thus, the possibility of its pro-oxidative activity getting hampered by the catalase upregulation by melatonin is minimised.

## 2. Material and Methods

The study was carried out on different healthy donors of both sexes who gave informed consent for the use of their blood samples for the study. The criteria for screening of volunteers included nonsmoking individuals having no acute or chronic diseases (such as diabetes mellitus, asthma, or tuberculosis) or organ dysfunction and had not taken any medication [8, 9]. The protocol of study was in conformity with the guidelines of the Institutional Ethical Committee. Blood samples were collected by venipuncture in heparinised vials (10 IU/mL) at two different timings of the day, namely, 10:00 hrs. (at the offset of melatonin secretion) and 22:00 hrs. (at the onset of melatonin secretion). The red blood cells (RBCs) were sedimented at 1800 g for 10 min at 4°C and washed three times with cold phosphate-buffered saline, pH 7.4 containing 0.154 mM NaCl and 10 mM Na_2_HPO_4_. Supernatant and buffy coat were carefully removed after each wash.

### 2.1. Determination of MDA Content

Erythrocyte MDA was measured according to the method of Esterbauer and Cheeseman with slight modification [10]. Packed erythrocytes (0.2 mL) were suspended in 3.0 mL PBS containing 0.5 mM glucose. The lysate (0.2 mL) was added to 1.0 mL of 10% trichloroacetic acid and 2.0 mL of 0.67% thiobarbituric acid, boiled for 20 minutes at temperature >90°C, cooled, and the absorbance read at 532 nm. Concentration of MDA is calculated using extinction coefficient (*ε* = 31,500) and is expressed as nmol·mL^−1^ of packed erythrocytes.

### 2.2. Determination of GSH Content

Erythrocyte GSH was measured following the method of Beutler [11]. The method was based on the ability of the –SH group to reduce 5,5′-dithiobis,2-nitrobenzoic acid (DTNB) and form a yellow coloured anionic product whose OD is measured at 412 nm. Concentration of GSH is expressed in milligram per millilitre packed RBCs and was determined from standard plot.

### 2.3. Induction of Oxidative Stress and In Vitro Effect of Melatonin

Blood was washed two to three times with PBS containing 5 mM glucose (GPBS), pH 7.4. Erythrocytes were then suspended in 4 volumes of GPBS. A stock solution (10 mM) of melatonin was prepared in absolute ethanol; further dilutions (10^−4^ M–10^−8^ M) were done with PBS. The concentration of ethanol was always <0.01% (v/v) in the final solution. The *in vitro* effect of melatonin was evaluated by incubating erythrocytes with melatonin at different doses (10^−5^ M–10^−9^ M final concentration) of melatonin for 30 minutes at 37°C. The erythrocytes were again washed two to three times with PBS, pH 7.4, to remove any amount of the compound, and finally, packed erythrocytes were used for the assay of MDA and GSH. In parallel control experiments, blood was incubated with ethanol (final concentration not more than 0.01% (v/v)) but without melatonin.

 Oxidative stress was induced *in vitro *by incubating washed erythrocytes with* tert*-Butyl hydroperoxide (10^−5^ mol·L^−1^ final concentration) in presence and absence of melatonin in the above experiments. The concentration of t-BHP used in the present study to induce oxidative stress of erythrocytes was in the range of concentrations used in other published reports [12].

## 3. Result and Discussion

The current study was designed to determine whether circadian rhythm-associated variations in the levels of malondialdehyde and glutathione occur in the erythrocytes of healthy adults and to investigate the concentration-dependent effect of melatonin on variation in these two biomarkers of oxidative stress. Malondialdehyde and hydroxynonenal, by-products of lipid peroxidation, are considered as toxic second messengers that can diffuse within or even escape from the cell and attack targets far from the site of production. MDA is considered to be one of the reliable markers of cellular peroxidative damage. 

**Figure 1:**
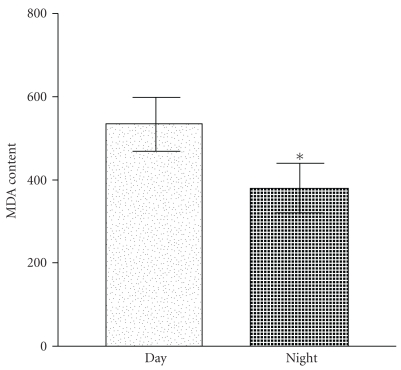
Day and night variation in malondialdehyde content in red blood cells. Significant decrease was observed in level of MDA in nocturnal samples (*P < .05). MDA content is expressed as nanomoles per millilitres of packed RBCs.

**Figure 2:**
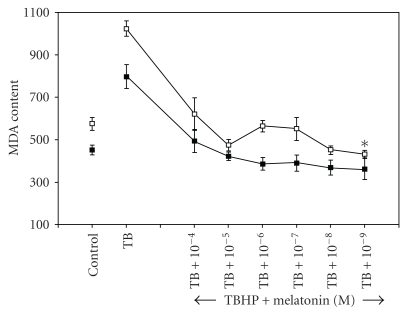
Day and night variation in malondialdehyde content in red blood cells after in vitro treatment with melatonin. The points (□) and (■) represent the effect of melatonin in diurnal and nocturnal blood samples, respectively. Significant variation was observed in diurnal-treated samples (*P < .05), the response being pronounced at nanomolar doses. MDA content is expressed as nanomoles per millilitres of packed RBCs.

Figure 1 shows a significant (*P* < .05) variation in erythrocyte MDA content in the blood samples collected during night and in the morning. Such a rhythmic variation in MDA is indicative of the relationship between oxidative stress and periodic changes in melatonin synthesis in response to photic and nonphotic signals. The dose-dependent effect of melatonin on erythrocyte MDA content after *in vitro* oxidative insult by incubating with t-BHP is shown in Figure 2. Incubation with t-BHP caused a significant increase in MDA content in erythrocytes obtained during day and night. A high dose of melatonin (10^−4^ M) resulted in a significant decrease in MDA content (*P* < .05), and lower concentrations of melatonin showed slightly greater protection of lipid peroxidation. It was observed that the physiological level (10^−9^ M) of melatonin exerted maximum protective effect. The effect of melatonin may be due to its free radical-scavenging property. 

**Figure 3:**
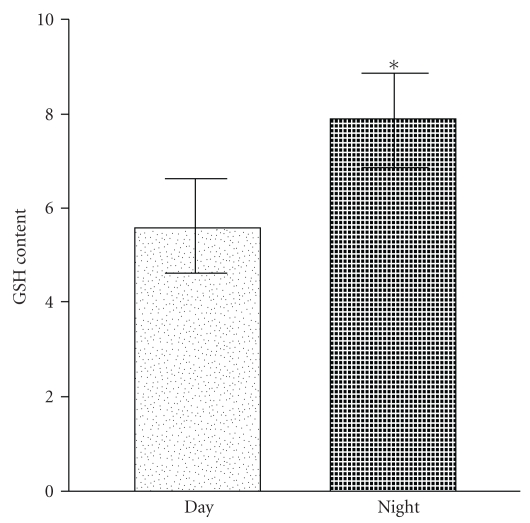
Day and night variation in intracellular reduced glutathione (GSH) content in red blood cells. Significant increase was observed in level of GSH in the diurnal samples (*P < .05). GSH content is expressed as milligram per millilitre packed RBCs.

**Figure 4:**
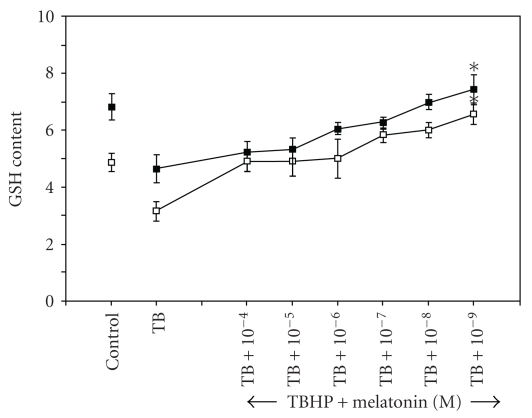
Day and night variation in reduced glutathione (GSH) content in red blood cells after in vitro treatment with melatonin. The points (□) and (■) represent the effect of melatonin in diurnal and nocturnal blood samples respectively. Dose-dependent variation was observed in both diurnal and nocturnal samples in response to *in vitro* treatment. Significant change was observed at 10^−9^ M concentration of melatonin (*P < .05). GSH content is expressed as milligram per millilitre packed RBCs.

Glutathione, an efficient antioxidant present in almost all living cells, is also considered as a biomarker of redox imbalance at cellular level.  Figure 3 shows marked increase in erythrocyte GSH content in nocturnal samples which highlights the role of endogenous melatonin in the circadian changes in cellular glutathione level. The dose-dependent increase in erythrocyte GSH content in the presence of melatonin can be seen in Figure 4. Exogenous melatonin demonstrated a protective effect against t-BHP-induced peroxidative damage in both diurnal and nocturnal samples, the effect being more pronounced at nanomolar doses. Melatonin was found to inhibit GSH oxidation in a dose-dependent manner; the antioxidative function of exogenous melatonin bears close proximity to peak physiological level of this indole neurohormone.

Oxidative free radicals resulting from cellular metabolism tend to disturb cellular membrane integrity and enzyme activities in red blood cells. Free radical-induced lipid peroxidation intensifies in the absence of a protective antioxidant defence mechanism resulting in decrease in cell membrane fluidity. Under physiological conditions, the reactive oxygen species are eliminated by enzymatic and nonenzymatic antioxidant defence systems. Knowledge of the absorption and metabolism of exogenous indoleamine drug becomes essential before any conclusions may be drawn regarding its potential to exert biological activity *in vivo*, as suggested by *in vitro* studies. Melatonin owes its free radical-scavenging property to the methoxy group at position-5 of the indole nucleus and the acetyl group of the side chain of melatonin [13].

The low concentration of MDA in nocturnal samples is in conformity with previous reports that proposed the periodicity in lipid peroxidation alongwith circadian rhythmicity [14]. Melatonin has been demonstrated to curb the cytotoxic effects induced by MDA [15]. Also, melatonin administration reduces lipid peroxidation in mammalian cells [16]. The effect of melatonin in combating the peroxidative degeneration was found to be more pronounced at a concentration close to nanomolar dose which may be underlying the earlier studies showing the nocturnal drop in cellular MDA content. The nocturnal blood samples, however, show no significant response to *in vitro* indoleamine treatment indicating suppressed activity of exogenous melatonin in presence of the endogenous hormone.

The observed decrease of lipid peroxidation cannot be explained merely on the basis of the direct scavenging of lipoperoxyl radicals by melatonin. The less effect of melatonin at a still higher concentration (10^−5^ M-10^−6^ M) may be attributed to the fact that the indoleamine is a weak lipoperoxyl radical scavenger and is least effective in counteracting the accumulation of lipid peroxides in the membrane of RBCs under induced oxidative challenge [17, 18]. Zavodnik et al. [19] reported inhibition of membrane lipid peroxidation in human erythrocytes treated with organic hydroperoxide as well as reduced radical-induced generation of luminal-dependent chemiluminescence after *in vitro* treatment with melatonin. Drug potency of melatonin at higher doses is quite evident from previous reports [20]. The protection of lipids by melatonin can also be explained as that of a preventive antioxidant in our system. The suppression of erythrocyte membrane lipid peroxidation by melatonin has prompted the suggestion for the use of melatonin for preventive and therapeutic purposes during cardiopulmonary bypass surgery [21].

The recycling of glutathione in the cells depends on an NADPH-dependent glutathione enzyme system which includes glutathione peroxidise, glutathione reductase, and *γ*-glutamyl-cysteine synthase forming a meshwork of an antioxidative system. Melatonin has been found to upregulate cellular glutathione level to check lipid peroxidation in brain cells [22]. The stimulatory effect of melatonin on the regulation of the antioxidant enzymes has been reported [23]. Since melatonin has an amphiphilic nature, its antioxidative benefit reaches the red blood cells, transcending the membrane barriers in a nonreceptor-mediated mechanism. Melatonin's antioxidative implications also extend to the upregulation of some antioxidant enzymes directly. Glutathione reductase and glutathione peroxidase, major constituents of the glutathione-redox system, are reportedly stimulated by melatonin [24]. The plasma GSH/GSSG redox state is controlled by multiple processes, which includes synthesis of GSH from its constitutive amino acids, cyclic oxidation and reduction involving GSH peroxidase and GSSG reductase, transport of GSH into the plasma, and the degradation of GSH and GSSG by *γ*-glutamyltranspeptidase. The increase in erythrocyte GSH concentration after melatonin administration could be linked to the known stimulation of *γ*-glutamylcysteine synthase, a rate-limiting enzyme in reduced glutathione synthesis, by melatonin [25]. The stimulation of GSH synthesis by melatonin is a major antioxidative action of melatonin. Experimental evidences are supportive of the circadian variation of cellular GSH levels [26]. The increase in the GSH level with exogenous melatonin in a concentration-dependent manner observed in our experiment signifies the efficacy of this indole compound close to normal physiological level.

## 4. Conclusion

Melatonin, due to its circadian secretion, causes day and night modulation in the markers of oxidative stress. Erythrocyte MDA and GSH contents are affected by melatonin according to the changes in photic signals, which emphasizes the role of melatonin as an antioxidant and its function against oxidative stress in red blood cells. The effect of exogenous melatonin has been related to plasma-membrane fluidity as well as its free radical-scavenging potential. In addition, exogenous melatonin has effects on these antioxidant-defence systems which may have important therapeutic implications.

## References

[1] Reiter RJ, Paredes SD, Manchester LC, Tan DX (2009). Reducing oxidative/nitrosative stress: a newly-discovered genre for melatonin. *Critical Reviews in Biochemistry and Molecular Biology*.

[2] Srinivasan V, Spence DW, Pandi-Perumal SR, Trakht I, Cardinali DP (2008). Therapeutic actions of melatonin in cancer: possible mechanisms. *Integrative Cancer Therapies*.

[3] Bonnefont-Rousselot D, Collin F (2010). Melatonin: action as antioxidant and potential applications in human disease and aging. *Toxicology*.

[4] Hardeland R, Tan DX, Reiter RJ (2009). Kynuramines, metabolites of melatonin and other indoles: the resurrection of an almost forgotten class of biogenic amines. *Journal of Pineal Research*.

[5] Pieri C, Marra M, Moroni F, Recchioni R, Marcheselli F (1994). Melatonin: a peroxyl radical scavenger more effective than vitamin E. *Life Sciences*.

[6] Domanski AV, Lapshina EA, Zavodnik IB (2005). Oxidative processes induced by tert-butyl hydroperoxide in human red blood cells: chemiluminescence studies. *Biochemistry (Moscow)*.

[7] Červinková Z, Křiváková P, Lábajová A (2009). Mechanisms participating in oxidative damage of isolated rat hepatocytes. *Archives of Toxicology*.

[8] Rizvi SI, Maurya PK (2007). Markers of oxidative stress in erythrocytes during aging in humans. *Annals of the New York Academy of Sciences*.

[9] Rizvi SI, Jha R, Maurya PK (2006). Erythrocyte plasma membrane redox system in human aging. *Rejuvenation Research*.

[10] Esterbauer H, Cheeseman KH (1990). Determination of aldehydic lipid peroxidation products: malonaldehyde and 4-hydroxynonenal. *Methods in Enzymology B*.

[11] Beutler E (1984). *A Manual of Biochemical Methods*.

[12] Di Simplicio P, Cacace MG, Lusini L, Giannerini F, Giustarini D, Rossi R (1998). Role of protein -SH groups in redox homeostasis—the erythrocyte as a model system. *Archives of Biochemistry and Biophysics*.

[13] El-Sokkary GH, Reiter RJ, Tan DX, Kim SJ, Cabrera J (1999). Inhibitory effect of melatonin on products of lipid peroxidation resulting from chronic ethanol administration. *Alcohol and Alcoholism*.

[14] Allegra M, Gentile C, Tesoriere L, Livrea MA (2002). Protective effect of melatonin against cytotoxic actions of malondialdehyde: an in vitro study on human erythrocytes. *Journal of Pineal Research*.

[15] Subramanian P, Mirunalini S, Pandi-Perumal SR, Trakht I, Cardinali DP (2007). Melatonin treatment improves the antioxidant status and decreases lipid content in brain and liver of rats. *European Journal of Pharmacology*.

[16] Livrea MA, Tesoriere L, D’Arpa D, Morreale M (1997). Reaction of melatonin with lipoperoxyl radicals in phospholipid bilayers. *Free Radical Biology and Medicine*.

[17] Tesoriere L, D’Arpa D, Conti S, Giaccone V, Pintaudi AM, Livrea MA (1999). Melatonin protects human red blood cells from oxidative hemolysis: new insights into the radical-scavenging activity. *Journal of Pineal Research*.

[18] Dikmenoglu N, Ileri E, Seringec N, Ercil D (2008). Melatonin prevents lipid peroxidation in human erythrocytes but augments deterioration of deformability after in vitro oxidative stress. *Clinical Hemorheology and Microcirculation*.

[19] Zavodnik IB, Domanski AV, Lapshina EA, Bryszewska M, Reiter RJ (2006). Melatonin directly scavenges free radicals generated in red blood cells and a cell-free system: chemiluminescence measurements and theoretical calculations. *Life Sciences*.

[20] Marchiafava PL, Longoni B (1999). Melatonin as an antioxidant in retinal photoreceptors. *Journal of Pineal Research*.

[21] Ochoa JJ, Vílchez MJ, Palacios MA, García JJ, Reiter RJ, Muñoz-Hoyos A (2003). Melatonin protects against lipid peroxidation and membrane rigidity in erythrocytes from patients undergoing cardiopulmonary bypass surgery. *Journal of Pineal Research*.

[22] Pandi-Perumal SR, Srinivasan V, Maestroni GJM, Cardinali DP, Poeggeler B, Hardeland R (2006). Melatonin: nature’s most versatile biological signal?. *FEBS Journal*.

[23] Reiter RJ, Carneiro RC, Oh CS (1997). Melatonin in relation to cellular antioxidative defense mechanisms. *Hormone and Metabolic Research*.

[24] Urata Y, Honma S, Goto S (1997). Melatonin induces gamma-glutamylcysteine synthetase mediated by activator protein-1 in human vascular endothelial cells. *Free Radical Biology and Medicine*.

[25] Blanco RA, Ziegler TR, Carlson BA (2007). Diurnal variation in glutathione and cysteine redox states in human plasma. *American Journal of Clinical Nutrition*.

[26] Martin M, Macias M, Escames G, Leon J, Acuna-Castroviejo D (2000). Melatonin but not vitamins C and E maintains glutathione homeostasis in t-butyl hydroperoxide-induced mitochondrial oxidative stress. *FASEB Journal*.

